# A role of inflammaging in aortic aneurysm: new insights from bioinformatics analysis

**DOI:** 10.3389/fimmu.2023.1260688

**Published:** 2023-09-06

**Authors:** Shilin Wang, Hao Liu, Peiwen Yang, Zhiwen Wang, Ping Ye, Jiahong Xia, Shu Chen

**Affiliations:** ^1^ Department of Cardiovascular Surgery, Union Hospital, Tongji Medical College, Huazhong University of Science and Technology, Wuhan, China; ^2^ Department of Cardiology, The Central Hospital of Wuhan, Tongji Medical College, Huazhong University of Science and Technology, Wuhan, China

**Keywords:** aortic aneurysms, inflammaging, ageing, inflammation, bioinformatics analysis

## Abstract

**Introduction:**

Aortic aneurysms (AA) are prevalent worldwide with a notable absence of drug therapies. Thus, identifying potential drug targets is of utmost importance. AA often presents in the elderly, coupled with consistently raised serum inflammatory markers. Given that ageing and inflammation are pivotal processes linked to the evolution of AA, we have identified key genes involved in the inflammaging process of AA development through various bioinformatics methods, thereby providing potential molecular targets for further investigation.

**Methods:**

The transcriptome data of AA was procured from the datasets GSE140947, GSE7084, and GSE47472, sourced from the NCBI GEO database, whilst gene data of ageing and inflammation were obtained from the GeneCards Database. To identify key genes, differentially expressed analysis using the “Limma” package and WGCNA were implemented. Protein-protein intersection (PPI) analysis and machine learning (ML) algorithms were employed for the screening of potential biomarkers, followed by an assessment of the diagnostic value. Following the acquisition of the hub inflammaging and AA-related differentially expressed genes (IADEGs), the TFs-mRNAs-miRNAs regulatory network was established. The CIBERSORT algorithm was utilized to investigate immune cell infiltration in AA. The correlation of hub IADEGs with infiltrating immunocytes was also evaluated. Lastly, wet laboratory experiments were carried out to confirm the expression of hub IADEGs.

**Results:**

342 and 715 AA-related DEGs (ADEGs) recognized from GSE140947 and GSE7084 datasets were procured by intersecting the results of “Limma” and WGCNA analyses. After 83 IADEGs were obtained, PPI analysis and ML algorithms pinpointed 7 and 5 hub IADEGs candidates respectively, and 6 of them demonstrated a high diagnostic value. Immune cell infiltration outcomes unveiled immune dysregulation in AA. In the wet laboratory experiments, 3 hub IADEGs, including BLNK, HLA-DRA, and HLA-DQB1, finally exhibited an expression trend in line with the bioinformatics analysis result.

**Discussion:**

Our research identified three genes - BLNK, HLA-DRA, and HLA-DQB1- that play a significant role in promoting the development of AA through inflammaging, providing novel insights into the future understanding and therapeutic intervention of AA.

## 1 Introduction

Aortic aneurysms (AA) have a high incidence globally, and only surgical treatment is an option once AA occurs. Approximately 13% of men and 6% of women aged over 65 have been diagnosed with abdominal aortic aneurysm (AAA) in the United States, with around 2 million new cases arising each year (1). The incidence of thoracic aortic aneurysms (TAA) is lower than that of AAA, but they are more challenging to manage (2). Nevertheless, the pathogenesis of AA remains unclear, and there is a scarcity of effective therapeutic strategies (3–5).

Ageing is a critical risk factor for the development of cardiovascular diseases (CVD) (6), and age-related CVD is one of the most significant issues worldwide (7). As an important type of CVD, AA typically occurs in individuals over 65 years old (3, 4). Moreover, persistent inflammation and immune cell infiltration are widely acknowledged as key mechanisms of AA development, which are observed in both plasma and local tissues of AA patients (8–12). Neutrophils and monocytes are recruited to the AA microenvironment as the initial step, followed by changes in cell phenotypes and activation of adaptive immune responses (13). Both ageing and inflammation play a crucial role in AA.

Interestingly, a growing body of evidence has shown that the ageing process is linked to chronic inflammation, characterized by elevated plasma inflammatory markers (14, 15), resulting in an increased risk of chronic diseases, notably CVD (16). Inflammaging, or age-related inflammation, plays a pivotal role in the development of chronic diseases and mortality in older individuals (10, 17, 18). Both ageing and inflammation are key pathogeneses of AA, but the interplay between them, inflammaging, has not yet been reported in previous studies on AA. Consequently, further investigation is required.

Bioinformatics is considered an effective method for identifying key molecules and investigating the potential molecular mechanisms of diseases by comparing differences between patients and healthy individuals using a variety of algorithms. In this context, we investigated the role of inflammaging-related genes in the development of AA and sought potential key genes involved in the related processes for further therapeutic targets.

## 2 Methods

### 2.1 Microarray data retrieval

Transcriptomic data files of AA were acquired from the public repository National Centre for Biotechnology Information Gene Expression Omnibus (NCBI GEO, http://www.ncbi.nlm.nih.gov/geo) (19) using “aortic aneurysm” AND “Homo sapiens” as search queries. The following filtering criteria were applied (1): all samples were from AA patients and non-AA controls (2); the sequencing type should be RNA-Seq (3); the sample size consisted of more than 15 samples (4); the test specimens were human aorta (5); data for the AA group were obtained from samples of non-Marfan atherosclerotic aortic aneurysms (6); there was no significant difference in age between the AA group and the normal group (7); the data was freely available for download and could be processed using all methods employed in the subsequent study. Based on these criteria, three datasets (GSE140947, GSE7084, and GSE47472) were obtained. The GSE140947 dataset (GPL18573 platform) included 12 AA and 12 normal human aorta samples, the GSE7084 dataset (GPL2507 platform) comprised 7 AA and 8 normal aorta samples, and the GSE47472 (GPL10558 platform), contained 14 AA and 8 normal aorta samples. In the stage of machine learning (ML), the GSE140947 dataset and GSE7084 dataset were used as training sets, while the GSE47472 dataset was used as the external validation dataset. Detailed information of the datasets used in the study is provided in **Supplementary Table S1**.

### 2.2 Data processing and differential gene screening

Significant differentially expressed genes (DEGs) from GSE140947 and GSE7084 were obtained using the R package “limma” respectively, with p-values < 0.05 and |log2 fold change (FC)| ≥ 1, where log FC > 1 represented upregulated genes and log FC < -1 represented downregulated genes. The heatmap and volcano plot of DEGs were generated using the R packages “pheatmap” and “ggplot2”.

### 2.3 Weighted gene co-expression network analysis

Weighted gene co-expression network analysis (WGCNA) was utilized to identify modules of highly correlated genes and their associations with disease phenotypes, in order to discover potential candidate biomarkers or therapeutic targets (20). All subsequent steps were based on R software (version: 4.2.2). We used WGCNA to identify AA-related modules, constructed using the R package “WGCNA” (20). After calculating the variance for each gene expression value, genes with absolute deviations greater than 25% from the median were extracted. Samples with outlier characteristics were excluded using the “goodSampleGenes” function (**Supplementary Figure S1**). The optimal soft threshold was chosen using the “pickSoftThreshold” function (**Supplementary Figure S2**), which was then used to construct a weighted adjacency matrix that was transformed into a topological overlap matrix (TOM). The soft threshold was 7 in both the GSE140947 and GSE7084 datasets. Subsequently, similar genes were categorized into co-expression modules using average linkage hierarchical clustering, with a minimum of 100 genes per module. Similar modules with a module eigengenes dissimilarity threshold (MEDissThres) < 0.2 were merged. To obtain modules related to clinical features, Pearson correlation analysis was employed. Finally, the modules with the strongest relevance to AA were selected for further analysis.

AA-related DEGs (ADEGs), identified by both WCGNA and the “limma” package, were obtained using the Jvenn online tool (https://jvenn.toulouse.inrae.fr/app/example.html) (21) by intersecting the results of the two methods.

### 2.4 Function enrichment analysis

To investigate the mechanism of ADEGs in AA, Gene Set Enrichment Analysis (GSEA) (22), Gene Ontology (GO) (23, 24), and Kyoto Encyclopedia of Genes and Genomes (KEGG) (25) pathway analyses were performed. The R package “org.Hs.eg.db” was used to obtain the Entrez ID for each ADEG, and “clusterProfiler” (26) was employed for biological function analyses. GSEA utilized the reference gene set “c2.cp.kegg.v7.4.symbols.gmt” (https://www.gsea-msigdb.org/gsea/msigdb/index.jsp). The results were visualized using the R packages “ggplot2” (27), “GOplot” (28), and “enrichplot”. In the three types of analysis, items with P < 0.05 in the Benjamini-Hochberg test were considered statistically significant. Physiological functions, including cellular component (CC), molecular function (MF), and biological process (BP), were incorporated in the GO analysis.

### 2.5 Identification of inflammaging and AA-related DEGs

The Human Gene Database, GeneCards (https://www.genecards.org/), was utilized to obtain inflammation and ageing-related genes by searching for the keywords “inflammation” and “ageing”. In order to obtain a reasonable number of genes, those with relevance scores above the median were selected. This resulted in 4416 genes associated with inflammation and 4302 genes related to ageing (**Supplementary Tables S2, S3**). By intersecting these gene sets, we identified 1070 genes related to inflammaging (**Supplementary Figure S3**; **Supplementary Table S4**). Inflammaging and AA-related DEGs (IADEGs) were revealed for further study using Jvenn, by intersecting the ADEGs from the two microarrays and the 1070 inflammaging-related genes.

### 2.6 Screening hub genes by protein–protein intersection network

To examine the interaction of proteins and their co-expression, the STRING database (https://cn.string-db.org/) (29) was employed to construct the protein-protein interaction (PPI) network. PPI networks with a minimum interaction score > 0.4 were then visualized using Cytoscape (version 3.9.1) (30). Cluster analysis was carried out using the CytoHubba and MCODE plug-ins in Cytoscape to identify significantly interacting genes. In the MCODE algorithm, with the following filter criteria: degree cut-off = 2, node score cut-off = 0.2, k-core = 2, max depth = 100, we selected the modules with the highest correlation. By implementing the MCC algorithm of the CytoHubba plug-in, 10 candidate hub genes were chosen. The intersecting genes of the two algorithms were used as the potential hub IADEGs for further wet laboratory experiments.

### 2.7 Screening hub genes by ML

ML was employed as another method to screen the hub IADEGs. Three ML algorithms – least absolute shrinkage and selection operator (LASSO), support vector machine (SVM), and random forest (RF) analysis – were adopted, and the overlapping genes among them were treated as potential hub IADEGs for further study. LASSO logistic regression analysis assigns a value of zero to the coefficients of variables one by one in order to identify highly important genes. SVM framework based on binary classification is a supervised ML technique that identifies the optimal model by maximizing the classification margin without overfitting and not depending on the number of samples. RF analysis is a type of ensemble learning based on decision trees, focusing on the score of each variable. SVM and RF were the two best ML methods before the invention of deep learning. The R package “glmnet” (31) was used for LASSO regression, while “e1071” (32) and “caret” (33) were used for SVM analysis, and “randomForest” (34) was used for SVM and RF analysis. The optimal lambda for LASSO regression was obtained through 100 resampling iterations of 10-fold cross-validation. Additionally, the processes of SVM and RF were also evaluated based on 10-fold cross-validations, and the same random number was used by the three types of analysis. The intersecting genes of the three ML methods were identified as potential hub IADEGs.

### 2.8 Nomogram construction and receiver operating characteristic evaluation

The clinical value of potential hub IADEGs was assessed using the area under the curve (AUC) and 95% confidence interval (CI) across three datasets, including two datasets for internal validation (GSE140947 and GSE7084) and one dataset for external validation (GSE47472). The R package “pROC” was employed to perform receiver operating characteristic (ROC) analysis and generate the AUC plots. A diagnostic value was considered when the AUC was above 0.75. The R package “rma” was utilised for the creation of a nomogram.

### 2.9 Prediction of a transcription factors mRNAs-miRNAs network

To explore the regulators of hub IADEGs, related transcription factors (TFs) and miRNAs were predicted. Three bioinformatics databases – starBase (35), TargetScanHuman (https://www.targetscan.org/vert_72/), and miRTarBase (36) – were employed to predict regulatory miRNAs for hub genes. MiRNAs identified by at least two databases were considered potential regulatory miRNAs. Key TFs of hub IADEGs were predicted using the Enrichr web server (37) with a p-value < 0.05. Finally, the linear TF-mRNA-miRNA coregulation network was visualized using Cytoscape.

### 2.10 Immune infiltration analysis

GSE7084 was utilized for immune infiltration analysis. The CIBERSORT algorithm (38) was employed to estimate the infiltration of immune cells in AA and normal aorta samples. Spearman correlation analysis was conducted to explore the relationship between various types of immune cells and potential hub IADEGs. The R packages “ggpubr” (39), “vioplot” (40), “corrplot” (40), and “ggplot2” (27) were used to visualize the results. The barplot and boxplot display the proportion of 22 types of immune cells in AA and normal aorta samples, while the violin plot shows the comparison between the two groups. The heatmap illustrates the association of immune cells. “Lollipop” plots were used to depict the correlation between hub IADEGs and immune cells.

### 2.11 Human specimen collection

Full-thickness aortic wall tissue was collected from patients undergoing aortic replacement operations due to AA (n=5) at Wuhan Union Hospital, Wuhan, China. AA specimens were gathered from the aneurysmal region of the aorta, whilst the normal samples were procured from the aorta of the recipient undergoing a heart transplant due to heart failure caused by ischemic cardiomyopathy or valvular disease. The clinical characteristics of the patients are listed in **Supplementary Table S5**. Specimens for qPCR and WB were snap-frozen in liquid nitrogen and stored at -80°C immediately. The samples used for immunohistochemistry (IHC) were soaked in a 4% paraformaldehyde solution. The collection of human aorta tissues and their use in our research were approved by the Review Board of Union Hospital Affiliated to Tongji Medical College, Huazhong University of Science and Technology, Wuhan, China. The research was carried out in accordance with the principles outlined in the Declaration of Helsinki. All enrolled patients provided written informed consent for the use of their aortic tissue for research.

### 2.12 RNA extraction and quantitative polymerase chain reaction

Total RNA was extracted from human aorta tissue using the RNA-easy Isolation Reagent (Vazyme, Nanjing, China), followed by quantitative polymerase chain reaction (qPCR) using HiScript III RT SuperMix for qPCR (Vazyme, Nanjing, China). Primers used in qPCR are listed in **Supplementary Table S6**, and synthesized by Sangon Biotech Co. Ltd. (Shanghai, China). The qPCR analysis was performed on StepOne Real-time PCR system (Applied Biosystems, Singapore). Relative changes in target gene expression were normalized to the expression levels of GAPDH, which were calculated using the 2^(-ΔΔCt)^ method.

### 2.13 Western blotting analysis

Total protein was isolated from human aorta tissue using RIPA lysis buffer and boiled in loading buffer for fifteen minutes. After separation on a 10% SDS-PAGE gel, the proteins were transferred to PVDF membranes and subsequently incubated with primary antibodies overnight at 4°C. The primary antibodies used in the study are listed in **Supplementary Table S7**. The membranes were then incubated with the HRP-conjugated secondary antibody (ABclonal Technology, Wuhan, China) for 1 hour at room temperature. The proteins were detected using a ChemiDoc XRS+ imaging system (Bio-Rad Laboratories, Hercules, CA, USA).

### 2.14 IHC

The primary antibodies used for IHC were listed in **Supplementary Table S7**. The tissue adhered on the slices were procedurally dewaxed, hydrated, and subjected to antigen retrieval. After incubation with 3% hydrogen peroxide solution for 25 minutes at room temperature, the slices were treated with 3% BSA for 30 minutes. The primary antibodies were used for an overnight incubation at 4°C, followed by culturing with secondary antibodies for 1 hour at room temperature. A DAB Detection Kit (Dako, California, USA) was used to stain the slide, and the cell nucleus was counterstained with haematoxylin.

### 2.15 Statistical analysis

Statistical analysis was carried out to analyze the results of wet laboratory experiment and the differences in baseline data of the included patients. All data included were presented as the mean ± standard deviation (SD) of the independent experiments. The differences were analyzed by Student’s T-test using SPSS 26.0. The expression level of genes in qPCR was calculated by Student’s T−test using GraphPad Prism 9.0. P<0.05 was considered to be statistically significant.

### 2.16 Refinement of the language

In order to improve the overall quality and readability of the article, we utilized a large language model, Chatbot Generative Pre - trained Transformer (ChatGPT, version 4, https://openai.com/blog/chatgpt), developed by OpenAI, to refine the language of our article (41, 42).

## 3 Results

### 3.1 Identification of DEGs by “Limma”

**Figure 1:**
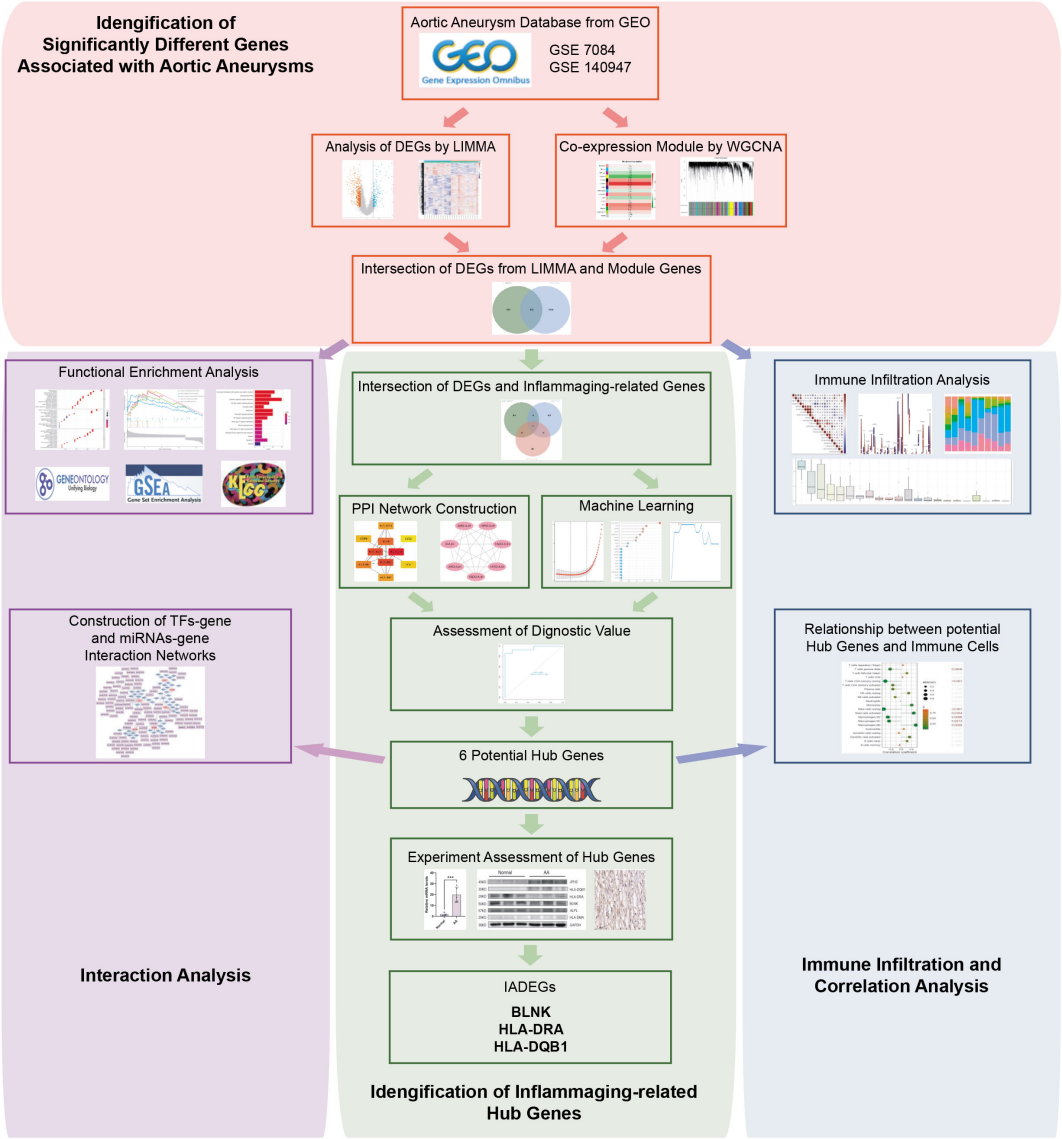
Flowchart of the study design and multi-step analysis strategy on bioinformatics data.

**Figure 2:**
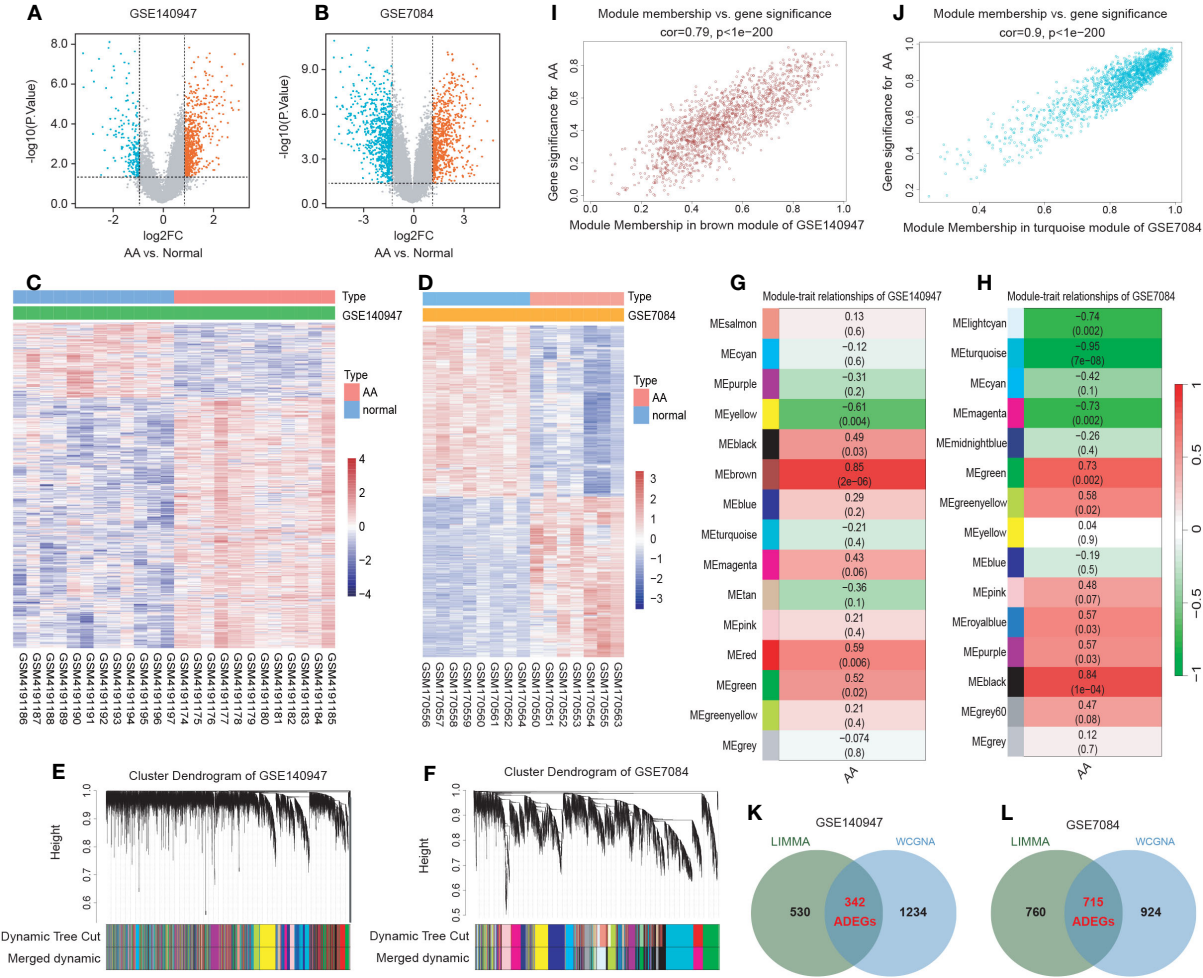
Identification of AA-related differentially expressed genes (ADEGs). **(A, B)** The volcano plots depict the significant differentially expressed genes (DEGs) identified by the “Limma” package based on the condition that p-values<0.05 and |log2 fold change (FC)| ≥ 1 from GSE140947 and GSE7084 datasets. Identified significant DEGs are highlighted in red (up-regulated genes) and blue (down-regulated genes). **(C, D)** The heatmap of significant DEGs identified from GSE140947 and GSE7084. **(E, F)** Gene co-expression modules represented by various colors under the gene tree. **(G, H)** The heatmap of the correlation between module genes and AA. The heatmap indicated that there were five modules significantly correlated with AA in the GSE140947 dataset and eight modules significantly correlated with AA in the GSE7084 dataset, under the condition of p-values<0.05. The brown module in GSE140947 and the turquoise module in GSE7084 demonstrated the highest correlation. **(I, J)** The correlation plot between the most significant module membership and gene significance of genes in the module indicated that the selected modules are closely associated with AA. **(K, L)** Venn diagrams display intersected DEGs from Limma and module genes among two datasets, which were identified as ADEGs.

The flowchart of the research procedure is displayed in **Figure 1**. Two GEO datasets related to AA were analyzed separately in this step due to the different GPL platform, resulting in a reduced number of genes after merging them. Using significance criteria, a total of 872 DEGs were obtained from GSE140947 dataset, comprising of 671 up-regulated genes and 201 down-regulated genes. GSE7084 dataset generated 1475 DEGs, including 714 up-regulated genes and 761 down-regulated genes. The DEGs obtained from both datasets using the “Limma” package are presented in **Supplementary Tables S8, 9**. The volcano plots and heatmaps of DEGs are illustrated in **Figures 2A–D**.

### 3.2 WGCNA and critical module identification

A scale-free co-expression network was created by WGCNA to identify the most associated module. After merging modules with high similarity (**Figures 2E, F**), the relationship between disease and genes in the finally generated module was assessed by Spearman correlation coefficient, which was visualized by heatmap (**Figures 2G, H**). The brown module in GSE140947 (1576 genes, r=0.85, p=2e-06) and the turquoise module in GSE7084 (1639 genes, r=-0.95, p=7e-08) had the highest correlation and were selected as key modules in the subsequent analysis. The genes contained in these modules are presented in **Supplementary Table S10**. **Figures 2I, J** demonstrate a significant correlation between module membership and gene significance (correlation coefficient = 0.79, p<1e−200 in GSE140947 dataset; correlation coefficient = 0.9, p<1e−200 in GSE7084 dataset), indicating that the selected modules are closely associated with AA.

### 3.3 Selection and functional enrichment analysis of ADEGs

The intersection of genes from Limma and module genes were obtained as ADEGs, as depicted in **Figures 2K, L**. 342 genes from the GSE140947 dataset and 715 genes from the GSE7480 dataset were acquired, and enrichment analysis for these genes was carried out. We conducted the functional enrichment of these shared genes from both datasets with GSEA, GO, and KEGG. The results of enrichment analysis are displayed in **Supplementary Tables S11–13**.

**Figure 3:**
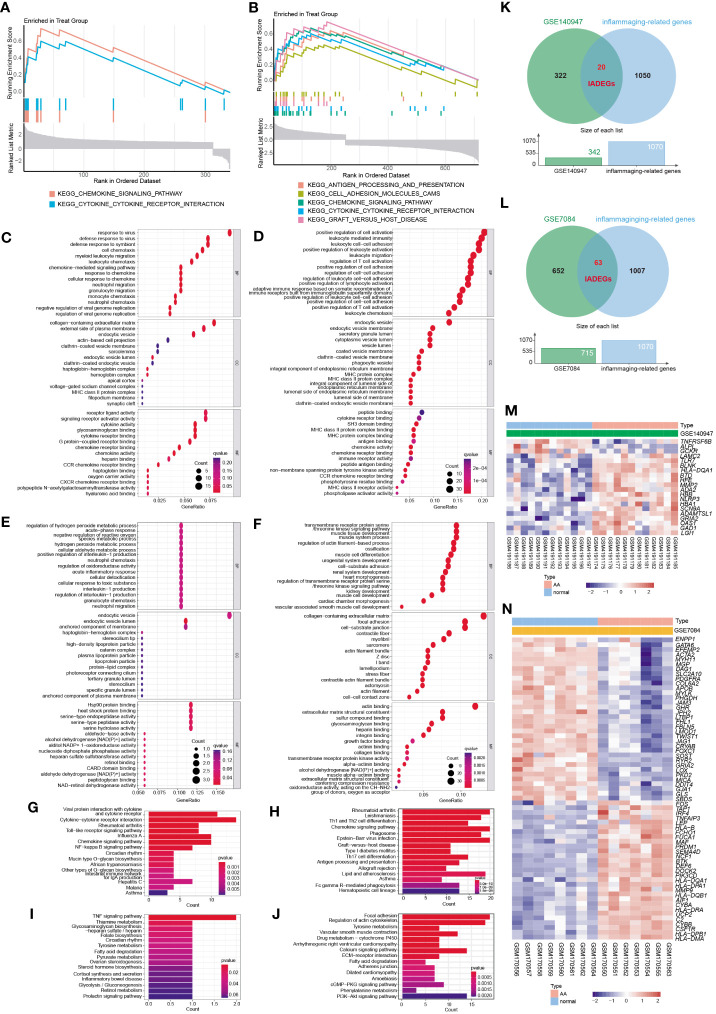
The functional enrichment analysis of ADEGs and the selection of inflammaging and AA-related DEGs (IADEGs). **(A, B)** GSEA analysis of the ADEGs revealed enrichment of pathways linked to chemokine signaling pathway, cytokine-cytokine receptor interaction, and cell adhesion. **(C-F)** GO enrichment of IADEGs in GSE140947 and GSE7084 datasets. **(C, D)** exhibited up-regulated expression pathways, whilst **(E, F)** displayed down-regulated expression pathways. (*BP* biological process, *CC* cellular component, *MF* molecular function) **(G–J)** KEGG enrichment of IADEGs in GSE140947 and GSE7084 datasets. **(G, H)** demonstrated up-regulated expression pathways, whilst **(I, J)** manifested down-regulated expression pathways. **(K, L)** The overlapping genes between ADEGs selected from GSE140947 **(K)** and GSE7084 **(L)** dataset and inflammaging-related genes were identified as IADEGs. **(M, N)** The heatmaps depict the differential expression of IADEGs in AA and normal tissue.

GSEA analysis of the ADEGs revealed enrichment of pathways linked to chemokine signaling pathway, cytokine-cytokine receptor interaction, and cell adhesion (**Figures 3A, B**). GO terms were categorized into Biological Process (BP), Cellular Component (CC), and Molecular Function (MF) as illustrated in **Figures 3C–F**. For BP, genes associated with “positive regulation of cell activation” and “chemokine-mediated signaling pathway” were up-regulated, while genes related to “regulation of hydrogen peroxide metabolic process” and “transmembrane receptor protein serine” were down-regulated. For CC, genes involved in “MHC protein complex” and “endocytic vesicle membrane” exhibited increased expression, while genes associated with “actin filament bundle” and “contractile fiber” displayed reduced expression. For MF, genes acting on “chemokine activity” and “peptide antigen binding” increased, while genes involved in “heat shock protein binding” and “actin binding” decreased. In KEGG analysis, the processes of “Cytokine-cytokine receptor interaction” and “Chemokine signaling pathway” were active, while “TNF signaling pathway” and “Focal adhesion” were suppressed (**Figure 3G–J**). In summary, the upregulated genes were enriched in pathways associated with inflammation and matrix remodeling, similar to the two major conserved up-regulated pathways in senescence (43), leading to higher levels of serum inflammatory markers (14, 15) and an increased risk of chronic diseases (16). Inflammaging, which is age-associated inflammation, is a process related to senescence and plays a crucial role in the development of chronic diseases in seniors (10, 17, 18). As a pathological process of chronic progression, AA is commonly observed in elderly people with elevated serum inflammatory markers and persistent inflammatory response in local tissues (9–12). Therefore, the next study mainly focused on the inflammaging process of AA.

### 3.4 Identification of IADEGs

To further explore whether and which genes could be associated with AA through inflammaging process, the intersection genes of ADEGs and inflammaging-related genes were identified as IADEGs as shown in **Figures 3K, L**. 20 and 63 genes were respectively identified as IADEGs from two datasets, displayed in **Figures 3M, N** and **Supplementary Table S14**.

### 3.5 Screening potential hub IADEGs via the PPI network

**Figure 4:**
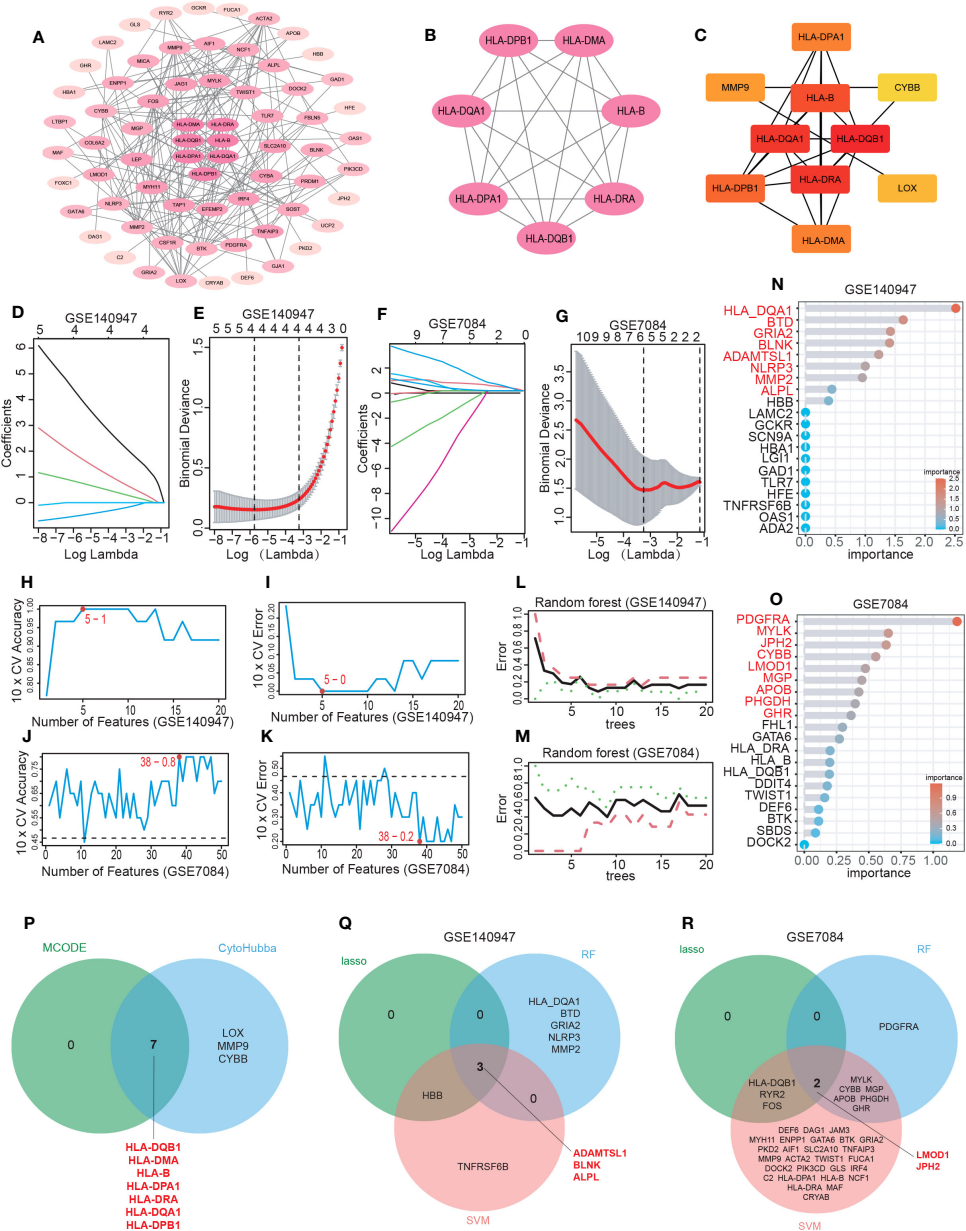
Hub IADEGs selection by PPI network and machine learning (ML). **(A)** The PPI network of IADEGs. **(B)** The PPI network of module genes with the highest score based on MCODE analysis. A key cluster with 7 genes was obtained. **(C)** Top 10 hub genes selected by MCC algorithm based on Cytoscape plug-in CytoHubba. **(D–G)** With the change of lambda value, the change trend of the coefficient corresponding to each observation value **(D, F)** and the selection of the best lambda value **(E, G)**. In **(E, G)**, the number of genes corresponding to the lowest point of the curve represents the most suitable lambda, which was 4 in the GSE140947 dataset and 5 in the GSE7084 dataset. **(H–K)** The variation of the accuracy and error of the SVM model with the number of features. The feature number with the highest accuracy, namely the lowest error, was selected for inclusion. 5 genes from the GSE140947 dataset and 38 genes from the GSE7084 dataset was selected for subsequent analysis. **(L–O)** Based on the minimum error shown in the plots of the error changing with the number of random trees, top 8 and 9 most important genes were selected from the GSE140947 dataset and the GSE7084 dataset. **(P)** The 7 intersecting genes identified by the two PPI-based methods. **(Q, R)** The 5 overlapping genes by three ML algorithms were selected respectively from two datasets as potential hub IADEGs for further assessment.

The PPI network was constructed using the String database and visualized using Cytoscape software, as depicted in **Figure 4A**. A module comprising 7 nodes and 21 edges was identified as the most significant module, as shown in **Figure 4B** and **Supplementary Table S15**. Utilizing the MCC algorithm of CytoHubba, the top 10 candidate node genes were selected from the PPI network, as displayed in **Figure 4C** and **Supplementary Table S15**.

### 3.6 Identification of potential hub IADEGs via ML

**Figure 5:**
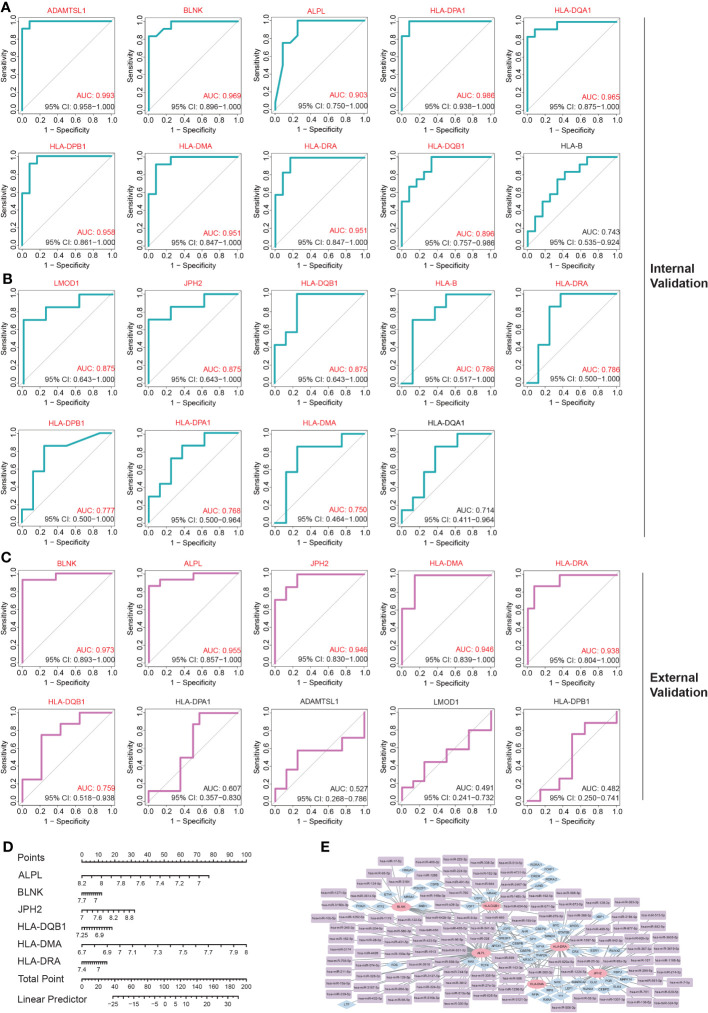
The diagnostic value assessment of each potential hub IADEGs and the hub IADEGs- transcription factors (TFs)- miRNAs regulatory network. **(A, B)** The receiver operating characteristic (ROC) curve of each potential hub IADEGs confirmed by internal validation sets including GSE140947 dataset **(A)** and GSE7084 dataset **(B)**. **(C)** The ROC curve of each candidate hub IADEGs validated in external dataset, GSE47472 dataset. **(D)** Nomogram for diagnosing AA with hub IADEGs in GSE47472 dataset. **(E)** The hub IADEGs-TFs-miRNAs regulatory network. The pink ellipse represents hub IADEGs, the blue diamond represents TF, and the purple rectangle represents miRNA.

The LASSO, SVM, and RF algorithms were employed to identify hub genes. Based on the optimal lambda in LASSO logistic regression (**Figures 4D–G**), 4 genes in the GSE140947 dataset and 5 genes in the GSE7084 dataset were recognized as candidate hub IADEGs genes (**Supplementary Table S16**). To minimize the classification error in the SVM model (**Figures 4H–K**), 5 genes in the GSE140947 dataset and 38 genes in the GSE7084 dataset were extracted from IADEGs (**Supplementary Table S16**). Based on the optimal number of trees with the lowest error rate (**Figures 4L, M**), the top 8 genes in the GSE140947 dataset and 9 genes in the GSE7084 dataset were obtained as potential hub IADEGs according to the importance in MeanDecreaseGini result (**Figures 4N, O**; **Supplementary Table S16**).

Ultimately, the overlapping genes of two PPI methods (**Figure 4P**) and three ML algorithms (**Figures 4Q, R**) were selected respectively in two datasets as potential hub IADEGs genes (**Supplementary Table S16**). The combination of these 12 hub genes was utilized for further assessment.

### 3.7 Diagnosis value evaluation of potential hub IADEGs

To further evaluate the diagnostic potential of these genes, three datasets, including the GSE140947 dataset, GSE7084 dataset, and GSE47472 dataset, were utilized for internal and external validation. As previously stated, a diagnostic value was considered significant when the AUC was above 0.75.

The internal validation demonstrated that 10 of these hub IADEGs candidates had AUC values > 0.75 in GSE140947 dataset or GSE7084 dataset, as illustrated in **Figures 5A, B**. HLA-B had an AUC value of 0.743 across the GSE140947 dataset, and HLA-DQA1 had an AUC value of 0.714 across the GSE7084, which had been excluded from hub IADEGs. Regarding the validation of the external dataset (shown in **Figure 5C**), 4 excluded genes had an AUC value below 0.75, including HLA-DPA1 (AUC=0.607), ADAMTSL1 (AUC=0.527), LMOD1 (AUC=0.491), and HLA-DPB1 (AUC=0.482). Therefore, BLNK, ALPL, JPH2, HLA-DMA, HLA-DRA and HLA-DQB1 were considered to have certain diagnostic value for AA and were identified as potential hub IADEGs for next wet laboratory experiments. Subsequently, a nomogram with the six hub IADEGs was constructed (**Figure 5D**).

### 3.8 TFs-mRNAs-miRNAs regulatory network construction

To investigate the upstream regulation of the hub IADEGs, a TFs-mRNAs-miRNAs regulatory network was established. Based on the Enrichr web server, 53 corresponding TFs were obtained with a p-value < 0.05 (shown in **Supplementary Table S17**). A potential 113 miRNAs were predicted by at least two bioinformatics databases, including starBase, TargetScanHuman, and miRTarBase database, as illustrated in **Supplementary Table S18**. **Figure 5E** presents the interaction between hub IADEGs and their regulatory TFs and miRNAs.

### 3.9 Immune infiltration analysis in AA

**Figure 6:**
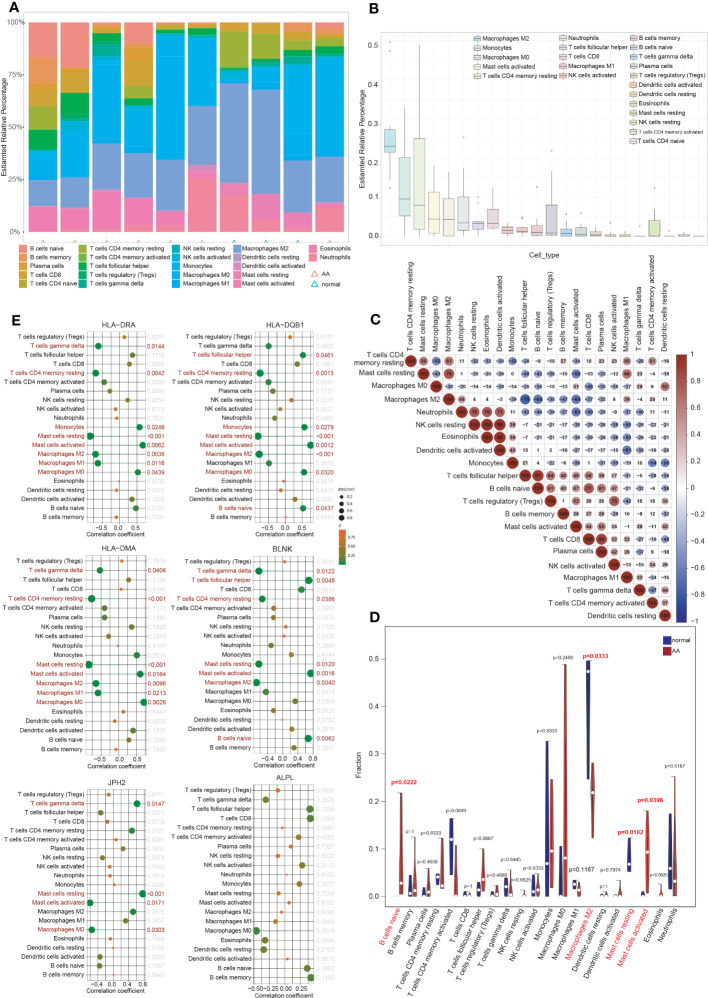
Immune cell infiltration analysis. **(A)** The proportion of 22 types of immunocytes between AA and normal groups visualized by the stacked histogram. **(B)** The percentage of immunocytes infiltrated in AA tissue. **(C)** Heatmap showed the multiple correlations between the infiltrating immunocytes in AA. **(D)** Violin plot showed the comparison of different kinds of immune cells between AA and control groups. Notable differences (P < 0.05) were observed in four types of immunocytes. **(E)** Lollipop plots showed the association of 6 hub IADEGs with infiltrating immunocytes.

Inflammatory response and immune regulation are components of the pathogenesis of AA (9–12), and the impact of immunity in AA can be better examined through immune infiltration analysis. To evaluate the significance of various immunocytes in the immune microenvironments of AA, the GSE7084 dataset was employed to assess the degree of their infiltration. The proportion of 22 types of immunocytes in AA and normal aorta tissue is depicted in **Figure 6A**. The percentage of immunocytes infiltrated in AA tissue is illustrated in **Figure 6B**, indicating that M2 type macrophages were the most abundant followed by monocytes and M0 type macrophages. The multiple correlations between the infiltrating immunocytes in AA are displayed in **Figure 6C**. For the markedly distinct cells, a robust interaction was observed between anti-inflammatory M2 type or proinflammatory M1 type macrophages and other immunocytes, which have a pivotal role in the progression of inflammation from tissue damage to tissue healing (8, 44). In **Figure 6D**, notable differences (P < 0.05) were observed between AA and normal groups in four types of immunocytes, specifically, naive B cells, M2 type macrophages, resting and activated mast cells. Multiple studies have indicated that the dysfunction of macrophage phenotypic transformation might be a result of ageing and related to a persistent elevated serum inflammatory response (45). In summary, the findings of the correlations between the infiltrating immunocytes demonstrated the suppression of inflammation and the repair of damaged tissue.

### 3.10 Relationship between hub IADEGs and immune cells

The association of 6 hub IADEGs with infiltrating immunocytes was illustrated in **Figure 6E**. Each hub IADEG was linked to the infiltration status of immunocytes, apart from ALPL. The up-regulated IADEGs, comprising HLA-DRA, HLA-DQB1, HLA-DMA, and BLNK, were positively correlated with monocytes, activated mast cells and follicular helper T cells, whilst they demonstrated a negative correlation with M1 and M2 type macrophages. JPH2, the down-regulated IADEGs, were primarily associated with M0 type macrophages and mast cells.

### 3.11 Experimental validations of hub IADEGs expression in AA patients’ aorta

**Figure 7:**
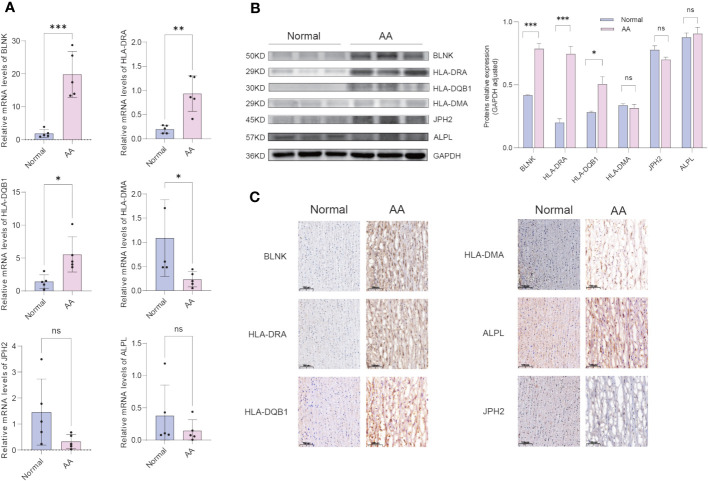
Confirmation of hub IADEGs expression in human aorta tissue. **(A)** Relative mRNA levels were detected by qPCR. **(B)** Representative western blot (WB) analysis (left) and quantification (right) of 6 hub IADEGs protein level in the human aorta tissue. **(C)** Immunohistochemical staining of 6 hub IADEGs in aorta sections of humans. (Scale bar, 100 μm). (ns: P > 0.05; *: P ≤ 0.05; **: P ≤ 0.01; ***: P ≤ 0.001).

The clinical features of the sample utilized are presented in **Table 1**. The expression of 6 hub IADEGs was validated in human aorta tissue using qPCR (**Figure 7A**). In comparison with normal aorta tissue, BLNK, HLA-DRA, and HLA-DQB1 exhibited significantly increased expression in the AA group, whilst HLA-DMA displayed a notable decrease. To further validate at protein level, western blotting (WB) and IHC were employed (**Figures 7B, C**). The findings indicated that the protein expression of BLNK, HLA-DRA, and HLA-DQB1 were in line with that of mRNA and the result of bioinformatics analysis. Taken together, the up-regulated expression of BLNK, HLA-DRA, and HLA-DQB1 in aorta tissues were strongly associated with AA.

**Table 1:** The clinical characteristics of the sample.

Feature	AA	normal	P value
Subjects (specimens)	n=5 (10)	n=5 (10)	–
Age (years)	59.00 ( ± 11.66)	48.40 ( ± 8.69)	0.1418
Males (N; %)	4 (80.00%)	4 (80.00%)	1.0000
BMI (kg/m2)	23.90 ( ± 2.58)	24.25 ( ± 2.10)	0.8199
Smoking (%)	4 (80.00%)	3 (60.00%)	0.4902
Diabetes mellitus (N; %)	2 (40.00%)	2 (40.00%)	1.0000
Hypertension (N; %)	5 (100.00%)	1(20.00%)	0.0098
Hyperlipidemia (N; %)	5 (100.00%)	4 (80.00%)	0.2918

The red value means P <0.05.

## 4 Discussion

AA frequently occurs in the ageing population over 65 years old (3, 4), accompanied by persistent elevated serum inflammatory markers (9, 10) and chronic inflammation of the aorta (11, 12), resulting in tissue damage and fibrosis. Utilizing a variety of bioinformatics techniques, ADEGs from two AA-related GEO datasets have been identified, enriched in pathways linked to inflammation and matrix remodeling, akin to the two major conserved pathways in senescence (43). Our study investigated the characteristics of inflammaging in AA based on bioinformatics analyses to pinpoint reliable biomarkers.

Several potential mechanisms contributing to ageing have been elucidated, including attrition of telomeres (46), oxidative damage (47), activation of the senescence-associated secretory phenotype (SASP) (48), et al. Our study focused on a recent addition to these mechanisms: inflammaging, namely, age-related inflammation. Previous studies have demonstrated that advancing age may be associated with higher-level basic systemic inflammation (14, 15), and evaluated serum inflammatory markers may contribute to an increased risk of chronic diseases (16). Age-associated inflammation, namely, inflammaging, plays a crucial role in driving chronic diseases, represented by CVD and death in elderly people (10, 17, 18). Based on the findings of existing studies, our research focused on analyzing the roles of inflammaging in the development of AA and exploring related targets. Our findings may help us better understand the crosstalk between AA and inflammaging, providing new insights into the pathophysiological processes of AA.

There is no consensus on the genes associated with inflammaging. We utilized GeneCards, the human gene database, to acquire inflammaging-related genes, and then employed PPI network and ML to obtain 6 hub IADEGs strongly correlated with AA. Small portions of PPI networks with highly connected regions possess a greater likelihood of being involved in biological processes, whilst those with fewer connections may not play a crucial role in the overall network’s integrity. Based on the degree of connectivity within the PPI network, the core genes identified and assessed were considered part of hub genes. ML, a significant branch of artificial intelligence, is capable of handling higher-dimensional feature data and has been extensively employed to acquire hub genes. In our study, the overlapping genes of three complementary ML algorithms - LASSO, SVM, and RF - were identified as another part of hub IADEGs, which may be more reliable than utilizing just one. ROC and nomogram were utilized to assess the diagnostic value of hub genes. Ultimately, 6 genes, including JPH2, BLNK, ALPL, HLA-DQB1, HLA-DMA, and HLA-DRA, were selected as hub genes after validation in two internal datasets and an external dataset. Further analysis of these 6 hub IADEGs revealed that 3 genes had significant co-expression tendencies, including BLNK, HLA-DRA, and HLA-DQB1, suggesting that these genes may play a significant role in promoting the development of AA through inflammaging process.

Some of the genetic determinants of ageing reside in polymorphisms in genes that regulate immune responses. It has been identified that ageing-related loci are associated with variants in the major histocompatibility complex region of chromosome six, coding human leukocyte antigen (HLA) proteins, based on a study of 164,610 UK individuals aged 60 to 70 years (49). HLAs are cell-surface proteins significant for the regulation of immune response and function, and the variation in specific HLA types is associated with several chronic diseases (50) and frailty (51) in elderly people. HLA-DQB1 is localized to the cell membrane with four functionally different variants associated with longevity (52). Cholesterol metabolism in liver cells could be affected by HLA-DQB1 by reducing the cytokines released by T cells, contributing to changes in plasma lipid homeostasis, which may be the mediating factor between HLA-DQB1 and longevity (52). The association between HLA-DQB1 and human longevity has been demonstrated by studies in Sardinia with a relatively isolated population, Okinawa, renowned for longevity, and centenarians in the Chinese population (52–54). Besides, HLA-DQB1 plays an important role in ageing-related chronic diseases, including age-related macular degeneration (55), ischemic stroke (56), neurodegenerative diseases (57–59), chronic back pain, and osteoporosis (60), as well as autoimmune diseases (61–63). HLA-DRA is a known marker of T cell activation, representing the activated state of the immune system, which up-regulates in organ transplantation rejection (64) and down-regulates after multi-trauma (65). The expression of HLA-DRA increases in the range of 60-69 years old, while decreasing after 70 (66), which may suggest changes in immune system function with age. HLA-DRA is also a hub DEGs in the process of normal brain ageing (67), frontotemporal dementia (68), and ageing periodontitis tissues (69). The HLA-DMA gene codes for one of two chains of HLA-DM, which is a non-classical MHC II protein and widely exists in various antigen-presenting cells (70). As a membrane protein, HLA-DM mediates antigen internalization of B cells and dendritic cells (70) and plays an important role in peptide-induced T cell activation (71). Genotyping of HLA-DMA may be associated with the susceptibility to systemic lupus erythematosus (SLE) (72) and the disease severity of rheumatoid arthritis (73).

BLNK is required to promote B cell maturation and is a necessary component of BCR signaling pathways (74). Mutations of the BLNK gene may cause a block in the development of B cells (74), leading to an accumulation of pre-B cells in the bone marrow and pre-B-cell leukemia (75). As a potential tumor suppressor, BLNK is widely studied and considered a biomarker in leukemia (76, 77). Beyond the field of oncology, BLNK may also promote chondrocyte injury, contributing to osteoarthritis (78), and be associated with the microglia response to amyloid-β, a pathological change of Alzheimer’s disease (79).

JPH2 is one of the four JPH isoforms expressed in excitable cells, including muscle cells and neurons, and plays a crucial role in cellular excitability by impacting ion channel function related to calcium on the plasma membrane and sarcoplasmic reticulum (80). Reduced expression of JPH2 in arterial smooth muscle cells may cause arterial hypercontractility, contributing to arterial dysfunction, such as hypertension (81). In cardiomyocytes, JPH2 promotes T-tubule development and T-tubule network maturation (80). Inherited mutations of JPH2 may cause hypertrophic or dilated cardiomyopathy (82–84), and loss of cardiac JPH2 levels may lead to heart failure and atrial fibrillation (85, 86).

In our study, the interaction between inflammaging and AA was explored through bioinformatics analysis. To the best of our knowledge, we obtained key genes of AA related to inflammaging processes and provided potential molecular targets for further study. We conducted a rigorous bioinformatics analysis. Three GEO datasets and two complementary methods of hub gene screening, PPI network and ML, were utilized in our study to avoid sampling and methodological bias. However, there are also several limitations. First, the hub genes have only been validated in human tissue, leading to a conclusion lacking the support of animal models and studies without homologous genes. Second, further experiments were not conducted to elucidate the molecular regulation mechanism of these genes and their specific roles in AA development and rupture *in vivo* and *in vitro*, which we will focus on in our subsequent studies. Third, our research is grounded in GEO datasets with a rather small sample size, which could impact the generalizability of ML models and make it challenging to draw extrapolative conclusions. In tasks with a small sample size and high dimensionality, the model trained using limited samples is prone to overfitting and underfitting the target tasks. Consequently, the performance gap between the actual trained model and the optimal model trained using existing features and algorithms cannot be effectively reduced through training and hyperparameter adjustment, leading to subpar overall model performance. To minimize the impact of this limitation, we employed a simpler model, SVM framework based on binary classification (87, 88), to prevent overfitting of the model. Additionally, we utilized two databases as the training set and chose the common genes from both databases as the IADEGs. We are of the opinion that with future technological advancements, ML techniques better suited for limited samples and high-dimensional data will emerge, enhancing the generalization capability of bioinformatics data models. Moreover, further bioinformatics analysis and experimental studies with larger sample sizes should be conducted to obtain crucial genes of specific biological processes of AA and elucidate its possible mechanisms and related pathways.

## 5 Conclusions

In summary, following an extensive bioinformatics analysis, 3 genes that play a crucial role in the pathological process of AA through inflammaging were identified and validated, including BLNK, HLA-DRA, and HLA-DQB1. Further research is required to elucidate the mechanisms of these genes in the pathological process of AA and to establish their clinical significance.

## Data availability statement

The original codes used for the analyses presented in the study are publicly available. This data can be found here: https://github.com/wangshilin2000/Source-code-for-figure-2-6-1260688-.

## Ethics statement

The studies involving humans were approved by Ethics Committee of Union Hospital Affiliated to Tongji Medical College, Huazhong University of Science and Technology. The studies were conducted in accordance with the local legislation and institutional requirements. The participants provided their written informed consent to participate in this study.

## Author contributions

SW: Conceptualization, Data curation, Validation, Writing – original draft. HL: Data curation, Formal Analysis, Methodology, Validation, Writing – original draft. PY: Data curation, Formal Analysis, Validation, Writing – original draft. ZW: Data curation, Formal Analysis, Validation, Writing – original draft. PY: Supervision, Writing – review & editing. JX: Supervision, Writing – review & editing. SC: Conceptualization, Supervision, Writing – review & editing.
