# Supplementary material for: A role of inflammaging in aortic aneurysm: new insights from bioinformatics analysis
Source: Front Immunol. 2023 Sep 6;14:1260688. doi: 10.3389/fimmu.2023.1260688 (PMC10511768; doi:10.3389/fimmu.2023.1260688)
Supplement: Supplementary file 2 [file DataSheet_1.pdf]

Supplementary Figures

Figure S1: Clustering dendrogram of GSE140947 and GSE7084 datasets in WGCNA analysis. 1

Figure S2: Identifying the soft threshold power in WGCNA analysis. 2

Figure S3: The overlapping genes between aging and inflammation, considered as inflammaging-related genes. 3

Supplementary Figure S1

A

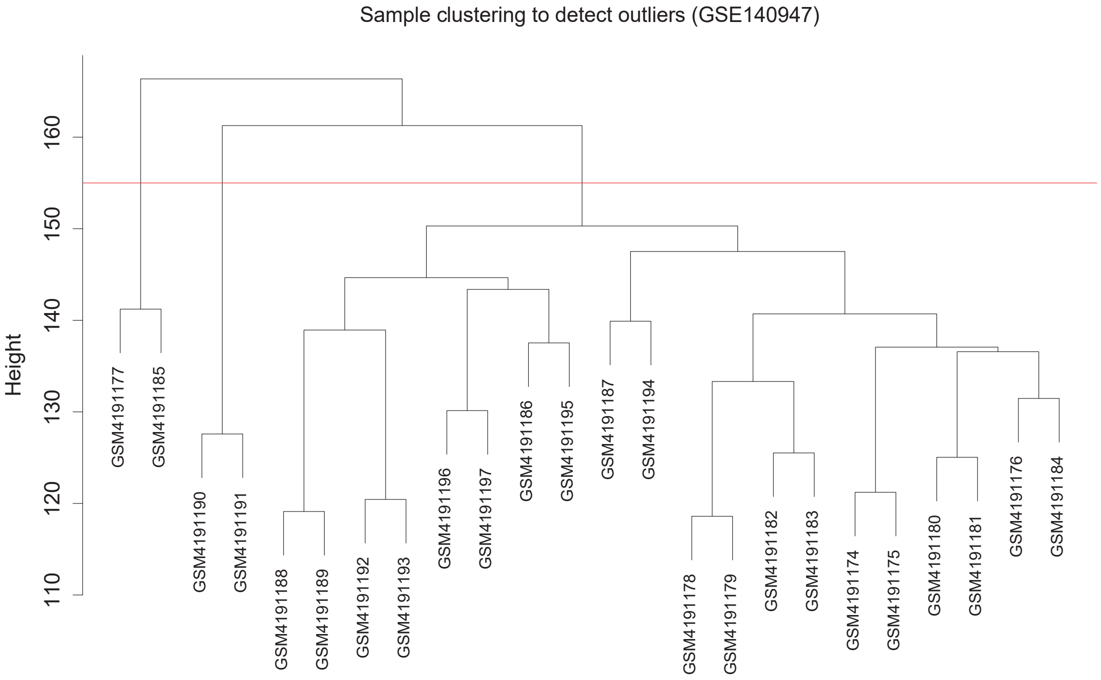

B

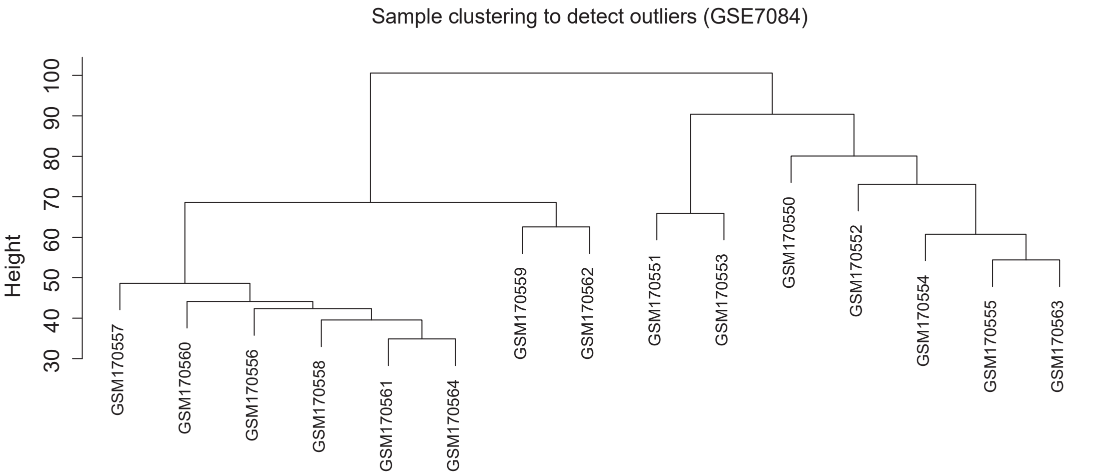

Figure S1: Clustering dendrogram of GSE140947 and GSE7084 datasets in WGCNA analysis. **(A)** GSE140947 dataset; **(B)** GSE7048 dataset.

Supplementary Figure S2

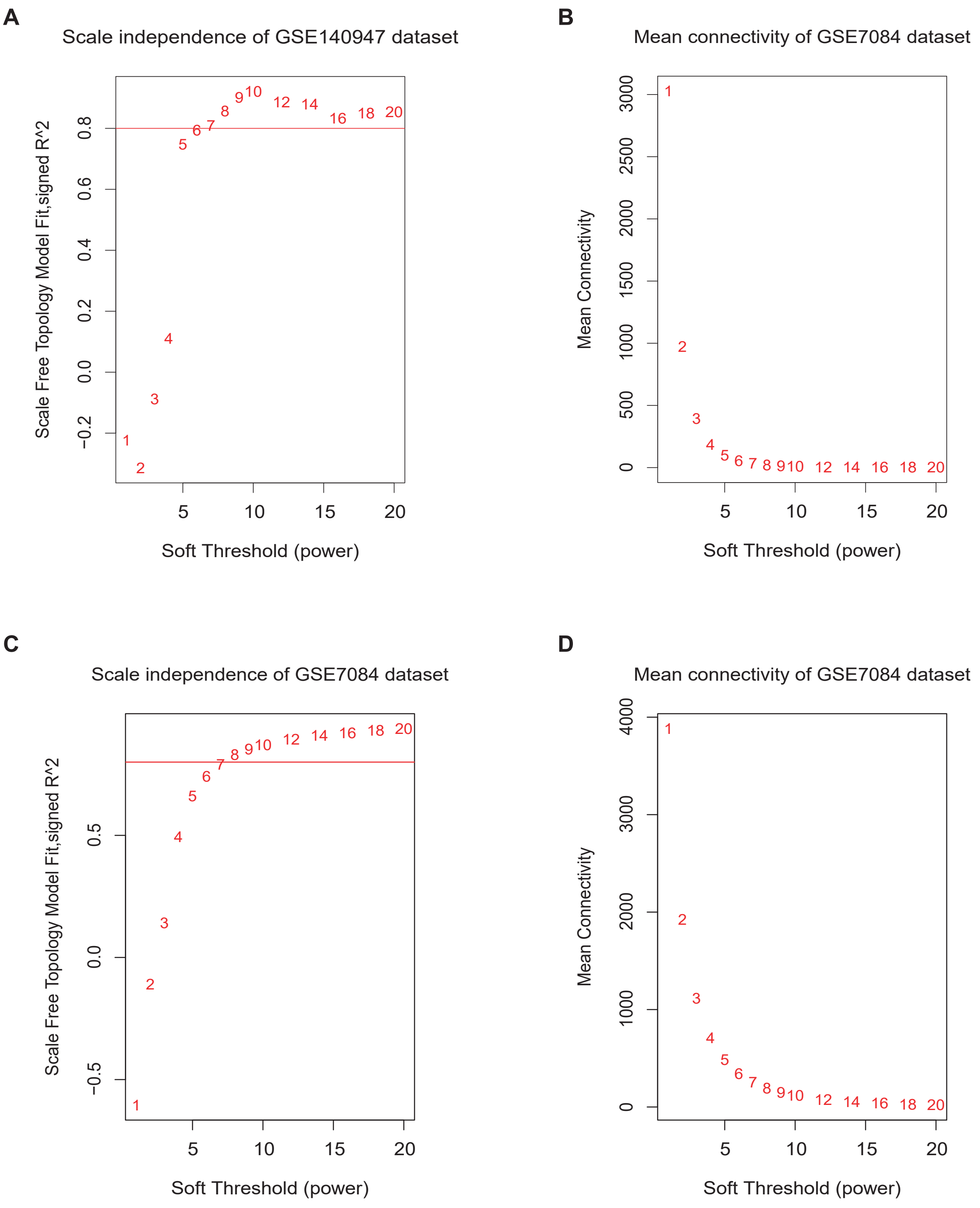

Figure S2: Identifying the soft threshold power in WGCNA analysis. The approximate scale-free fit index is achieved at the soft threshold power of 7 for both GSE140947 **(A.B)** and GSE7084 **(C,D)** datasets.

Supplementary Figure S3

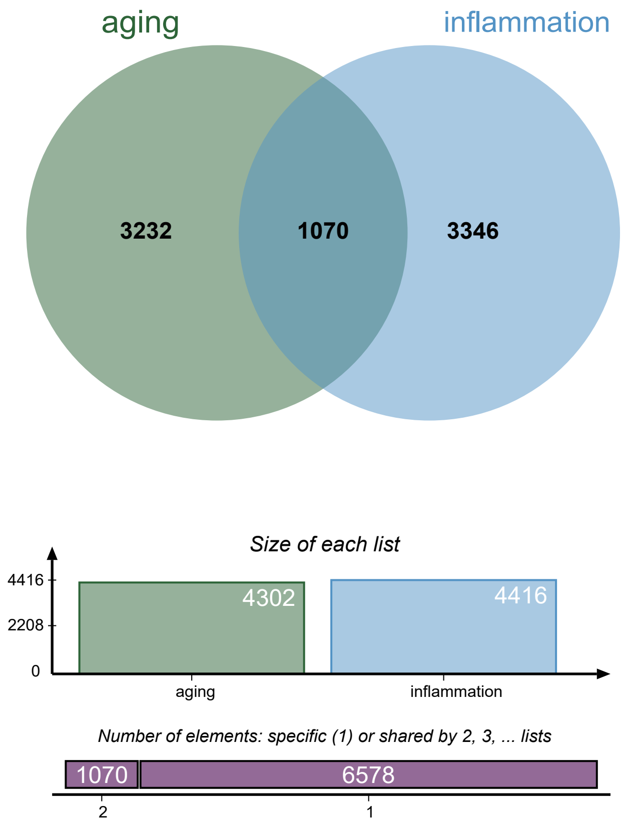

Figure S3: The overlapping genes between aging and inflammation, considered as inflammaging-related genes.
